# True inner self or fantasies and fabulation? Discourses on gender identity, sexuality, and LGBTQ among care workers in Swedish dementia care

**DOI:** 10.1177/14713012251319821

**Published:** 2025-02-13

**Authors:** Linn J Sandberg, Anna Siverskog

**Affiliations:** School of Culture and Education, Södertörn University, Sweden

**Keywords:** LGBTQ, dementia, sexuality, gender identity, dementia care

## Abstract

Lesbian, gay, bisexual, trans and queer (LGBTQ) people make up a considerable proportion of those in need of formal dementia care. Yet sexuality and gender identity have received little attention in dementia care research. Using a discursive approach, this article explores how dementia care workers discuss and reflect on dementia, gender identity, sexuality and caring for LGBTQ people with dementia. The article is based on a focus group study with dementia care workers in Sweden, primarily nurses and nurse assistants. The findings point to pervasive heteronormativity in everyday care practice; non-normative sexualities and gender nonconformity were primarily deemed invisible but also sometimes questioned as “fabulations” or “fantasies”. The invisibility of LGBTQ people was discursively framed as a result of generational belonging: people with dementia belonged to generations who were assumed to be closeted. Gender identity and sexuality were also framed as sensitive issues that were difficult to address with people with cognitive conditions. Dementia was understood as bringing out the true sexual or gendered self, but also as causing confusion and inauthentic expressions of gender and sexuality. In conclusion, existing discursive framings locate problems of heteronormativity outside of care practice and risk leading to inaction; more direct challenges to heteronormativity are needed in dementia care.

## Introduction

In recent decades there has been a considerable growth of research on the needs of older lesbian, gay, bisexual, transgender and queer (LGBTQ) people, including experiences of social services and care. Research suggests that older LGBTQ people expect to be in greater need of formal care and support than their heterosexual counterparts because they lack, to a greater extent, familial support and caregiving (Henning-Smith, Gonzales, & Shippee, 2015) Existing studies also indicate that older LGBTQ people are at higher risk of cognitive impairment due to factors such as minority stress, a lack of material and psychosocial support from a marriage relationship, and a higher prevalence of depression (Hsieh et al., 2021; Liu et al., 2021). Hence, although cognitive impairment does not equate with dementia, aging LGBTQ people face adversities which may also increase their likelihood of developing dementia. It is not clear what proportion of current older LGBTQ people are in need of formal care, but as the existing research discussed above indicates, professionals in dementia care are likely to provide care to this group at some point in their careers.

Moreover, the limited empirical research that exists on LGBTQ people’s experiences of living with dementia points to their significant worries and fears, both of losing social support and networks as their illness progresses and of encountering discrimination and heteronormativity in dementia care (Smith et al., 2024, Barrett et al., 2015; McParland & Camic, 2018, Anderson et al., 2021; Price, 2010; Silverman & Baril, 2023, Dykewomon, 2018). Overall, LGBTQ people with dementia in need of formal care are faced with particular and compounded forms of vulnerabilities, linked to both their cognitive illness and non-normative gender and sexual identities (Shippee et al., 2024; Westwood, 2016). There is thus a need for research on how formal dementia care professionals reflect on care provision for LGBTQ people with dementia and how gender identity and sexuality is considered in everyday care practice.

The scant attention to gender and sexuality in research on dementia care may seem surprising considering the almost hegemonic position of person-centred approaches in present-day healthcare policy, practice and research (Katz & Leibing, 2023). However, we suggest that this is explained by the liberal-humanist genealogies of person-centred approaches, which are largely ‘power silent’ (Grant, 2016). Critical dementia scholars have also noted that the experience of dementia at the intersections of gender, sexuality, race, class, age, and ability are very often overlooked and that dementia studies tend to shy away from discussions of systemic inequalities and oppression, including questions of heterosexism, homo- and transphobia, and hetero- and cisnormativity (Hulko, 2004; Sandberg, 2018; Sandberg & Ward, 2023).

In our work we thus seek to align with existing critical scholarship in dementia studies and interrogate how gender identity and sexuality discursively matters in dementia care and the implications its presence may have for people living with dementia. Building on five focus groups with 31 professionals working within Swedish dementia care, the aim of this article is to explore discourses on gender identity and sexuality in everyday care practice. Specifically, we focus on the articulations of dementia care workers and how they discuss and reflect on the intersections of dementia, gender identity, sexuality and caring for LGBTQ people with dementia.

The questions we explore in this article are:

- How are LGBTQ people, non-normative sexualities and gender nonconformity understood and discursively positioned in dementia care?
- How are dementia illnesses employed in discourses of sexuality and gender identity in dementia care?

The epistemological starting point of our study is discursive. We understand discourses on gender and sexuality in dementia care, articulated through policies, guidelines and everyday communications, as effectively shaping what subjects and practices become desirable, intelligible and possible. Our focus in this article is on neither attitudes nor what care workers effectively do. Instead, we are interested in the functioning of the articulations of dementia care workers, including what they position as problems and solutions and how they in turn may shape the ways care is practiced.

This article begins with a description of the Swedish context for this study. It then moves on to describe the study’s parameters, including the selection of participants and how focus groups were conducted, before outlining the theoretical and methodological framework. The analysis of the results which follows is divided into five sections before finally leading on to a concluding discussion.

## Dementia care and LGBTQ ageing in a Swedish context

Long term care for people with dementia in Sweden is the responsibility of the municipalities (Sw. *kommuner*) while medical care is provided by the administrative regions (Sw. *regioner*). The majority of older people with dementia in need of formal care are cared for in their homes by municipal home care services. For those who can no longer live on their own, Sweden offers residential care, called “special housing” (Sw. *särskilt boende*), where people living with dementia are cared for in special dementia units with 8–10 residents. However, since Swedish municipalities vary significantly in size and population there is great variation in the provision of specialised dementia care units (Jönson & Szebehely, 2018). Overall, a large proportion of people in residential eldercare in Sweden are estimated to live with dementia (Björk et al., 2016). Elder and dementia care in Sweden is universal, tax financed and provided after a needs assessment by municipal care managers.

The Swedish national guidelines for dementia care (Socialstyrelsen, 2017), state that the best care practice is person-centred and is defined as “focusing on the person with dementia” and “providing them with a personal care and care setting” (p.7, translations by authors). The care being offered “needs to depart from the person [with dementia’s] own wishes and needs and remaining capacities” (p.9). Gender identity and sexuality, however, are not mentioned specifically in the national guidelines. General discussions on the living conditions or special needs of LGBTQ people with dementia remain largely absent in Swedish dementia care policies, both on a local and national level (for further discussion on policies see (Siverskog & Sandberg).

Sweden is ranked high in terms of the civil rights afforded to LGBTQ people. In an international comparison, the country is positioned as progressive and tolerant in terms of sexual rights (ILGA, 2023). However, in line with other international research, existing qualitative studies on ageing LGBTQ people in Sweden identify the fears and concerns they have regarding their future care needs and their ability to advocate for themselves in formal care situations (Bromseth & Siverskog, 2013; Löf & Olaison, 2018; Siverskog, 2016, 2023). A recent inventory of the ways Swedish municipalities are working with older LGBTI people’s care needs, conducted by the National Board of Health and Welfare (Socialstyrelsen, 2023), concludes that there is a lack of knowledge of and systematic approaches to LGBTI issues in Swedish eldercare^1^. Less than one third (28%) of Swedish municipalities report that they mention LGBTI people in their guiding documents for eldercare. The inventory also revealed considerable variation between municipalities in terms of education on these issues.

The research literature on the health and social care of older LGBTQ people in Sweden and other Nordic countries is scarce (and non-existent when it comes specifically to dementia care) (Jakobsen et al., 2023). However, one recent scoping review focusing on the provision of healthcare services to older LGBT adults in the Nordics (Jakobsen et al., 2023), indicates that healthcare professionals lack sufficient knowledge and that older LGBT adults encounter hetero- and cisnormativity in the healthcare sector. Moreover, a recent inventory of healthcare, social welfare and social science study programmes by Bromseth and Siverskog (2023) shows how the presence of LGBTI issues are largely absent. Although professionals were generally supportive of the principle of equal treatment of LGBTI people there were significant knowledge gaps. Treating everyone equally tended to sustain heteronormativity and the invisibility of LGBTQ people (for similar international findings see e.g. Villar et al., 2015; Simpson et al., 2018). Although existing research on LGBT and eldercare in the Nordics does not focus specifically on dementia care, international research suggests that dementia care professionals have little knowledge of or competence with LGBTQ people (Nowaskie & Sewell, 2021; Shippee et al., 2024). It is likely that similar conditions also apply in Swedish dementia care.

All on all, while Sweden is relatively progressive in terms of LGBTQ rights and provides a relatively high standard of universal healthcare and social services, existing research suggests that person-centred elder and dementia care does not extend to questions of gender and sexuality on a policy level. Hetero- and cisnormativity pervades the Swedish healthcare and social services, with a detrimental impact on the lives of older LGBTQ people in need of dementia care (Siverskog & Sandberg).

## Methodology, study design and theoretical framework

The study discussed in this article was part of a wider research project on LGBTQ in Swedish dementia care, which also involved analyses of policies and guidelines (Siverskog & Sandberg) and interviews with LGBTQ people with dementia and their significant others (see Sandberg & Siverskog, 2024). This article examines the focus group interviews carried out with dementia care workers and how they articulated their experiences and thoughts on gender, sexuality and LGBTQ people with dementia.

The focus groups were recruited through contacting and presenting the study to different municipalities in Sweden and by presenting the study at the annual Swedish Dementia Days. We included only people who were currently working in dementia care and prioritised frontline care workers involved in day-to-day personal care. In some municipalities care managers and organisational developers were used as gate keepers for access to participants or as the catalyst for setting up a local focus group. These gatekeepers were often motivated to help us because they perceived a need for further knowledge of LGBTQ issues among their staff. As a recompense for their time and effort in participant recruitment, since the focus groups have completed we have carried out guest lectures in the municipalities and distributed copies of our publications. Although these gatekeepers provided us with essential access to participants, their choices determined who joined the study; we had little to no insight into their selection process. However, overall, it seems likely that people with more tolerant and positive attitudes to gender and sexual diversity agreed to take part.

All in all we recruited five focus groups with 31 participants in total. The majority of participants were either nurse assistants (13) or registered nurses (14) and some were specialised in dementia and dementia care. Among the participants were also two medical doctors and two occupational therapists. All of the participants had experience of caring for people with dementia in residential care and homecare services. Some participants were currently working in a memory clinic and others were employed as specialist nurses in dementia teams, which involved activities such as supporting dementia care staff in ethically challenging situations and organisational development. Some of these participants had an interest in and had been working with sexual health in dementia care and had in some cases heard lectures and read educational materials developed by us (Sandberg & Larsdotter, 2022a; Siverskog, 2021). The composition of the groups varied. In some cases the participants worked in the same workplace while in others the groups consisted of a mix of people from different municipal workplaces. All focus groups were mixed in terms of professions. Participants were aged 31–65 and had been working with elder and dementia care for3– 44 years (average 20 years). Participants were predominantly women (28) and a quarter (8) had migrated to Sweden, a proportion which roughly reflects the ethnic composition of the contemporary Swedish dementia workforce .

We find focus groups to be useful because they, in the words of Wilkinson (1998, p. 112), enable studies of “the co-construction of realities between people” and “the dynamic negotiation of meaning in context”. We thus understand focus groups to have some similarities with how meaning is negotiated in dementia care, for example, in everyday conversations, meetings and coffee breaks. Focus groups lasted between 120 and 180 minutes and were recorded and transcribed verbatim. Three focus groups were led by both authors, Sandberg and Siverskog, and two were led by Sandberg only. During the first part of each focus group meeting we asked questions about sexuality and gender identity. We asked whether or not sexuality and gender identity figured in their day-to-day dementia care work, how these topics presented themselves, and what their experiences were of caring for LGBTQ people with dementia. During the second part of each focus group we used six short vignettes to elicit further discussion. The vignettes, which were distributed on paper to all participants and read out loud, involved different cases with LGBTQ people with dementia in need of formal care, often in ethically challenging scenarios (see Appendix A for vignettes). We chose to use vignettes because we surmised, based on previous research, that some interviewees would be likely to claim that they had never encountered LGBTQ persons in eldercare (compare Willis et al., 2016; Villar et al., 2022; Sandberg & Larsdotter, 2022b) but we still wanted to hear more about how they might reflect on same-sex desire and relationships as well as gender nonconforming identities/expressions in day-to-day dementia care. Participants were initially asked: What are your reflections on this situation? What would you do? The vignettes were fruitful because they produced a wide range of reactions and responses and revealed varying and sometimes conflicting articulations.

The project has been reviewed and approved by the Swedish Ethical Review Authority (dnr 2022-00886-01). All participants were asked for their informed consent. Still, we understand research ethics as going beyond ethical protocols and as fundamentally intertwined with methodology. Consequently, we see the conversations in the focus groups as shaped in relation to networks of power and inequality, which operate both in the direct interactions between participants and in relation to wider societal discourses. Elder and dementia care workers in Sweden, as in many places across the globe, often work under stressful conditions and may be precarious in terms of work security. Thus, the participants may have felt the pressure to perform as “good care workers” in the focus groups and their responses may have been shaped in relation to both societal discourses of “crisis in care” and the fact that may have been asked by their care managers to take part. Also, that some participants worked together may have shaped how and what was articulated. To create a secure environment, we thus repeatedly stressed the confidentiality and anonymity of everything that was said in the interviews and the value of different views. Moreover, in groups that were mixed in terms of race and ethnicity there were examples of how the white participants with Swedish backgrounds were more assertive and also occasionally asked other participants with non-Western backgrounds about their attitudes to homosexuality, thus reflecting underlying assumptions in Sweden of migrants as intolerant Others (Kehl, 2020; Polkov, 2024).), Also in terms of the sexualities and gender identities of care workers, none of the participants were open about identifying as LGBTQ (and neither were we as researchers). This may have contributed to the prevailing heteronormative views in the focus groups.

As stated in the introduction, our epistemological starting point is discursive. We regard this post-structuralist approach as both a theoretical and a methodological framework (Winther Jørgensen & Phillips, 2002). More specifically we are inspired by the concepts and terminology developed in discourse psychology and as formulated by the work of Wetherell and Potter (Potter & Wetherell, 1987; Wetherell & Potter, 1988). This approach derives its inspiration both from ethnomethodology and conversation analysis with the focus on active meaning-making on the micro-level (in everyday talk and conversation), and discourse theories on a macro-level (how people’s articulations are also situated in relation to a wider discursive terrain of power relations). To understand the articulations of dementia workers we use the concept “interpretative repertoire”. As described by Wetherell and Potter (1988, p. 172) interpretative repertoires “can be seen as the building blocks speakers use for constructing versions of actions, cognitive processes and other phenomena”. Discourses are thus formed out of interpretative repertoires which are often contradictory and varied. We are interested in the function of different kinds of speech in the focus groups we have conducted, what is “at stake” and what is accomplished through the different ways participants make sense of the intersections between gender identity, sexuality, LGBTQ, and dementia. Importantly, even though discourses have practical consequences they are not always explicit or indeed intentional. In no way do we wish to suggest that the participants in our study were deliberately trying to excuse or justify themselves in the focus groups. Our aim is to go beyond individual sensemaking and instead to stress how all articulations exist in particular “rhetorical contexts” (Billig, 1996). Some things are possible to articulate but others can be understood as unintelligible or outside of existing discursive frameworks.

The analytical process undertaken to outline the existing interpretative repertoires first involved coding the material, with the two authors initially coding separately and then discussing and comparing the codes together. The codes were then re-examined and potential interpretative repertoires mapped out, with a particular focus on contradictions and variations. Inspired by Laurel Richardson’s (1994) notion of “writing as a method of inquiry”, the analyses continued through the later stages of writing and rewriting.

The discourse theoretical framework that we employ in this article is complemented by concepts and understandings derived from queer theory which, similar to discourse theory, has its genealogy in post-structuralism. Instead of focusing on so called sexual and gender minorities, queer theory provides a post-realist and post-positivist framework for interrogating normativity and power related to gender and sexuality (for further discussions on queer theory and dementia studies see King, 2016, 2023). Of central importance is the concept of heteronormativity which denotes the naturalisation and privileging of heterosexuality and gender-conforming subjects or cis people (Butler, 1990; Warner, 1993).

## Findings

### “They belong to the old times”: A generation of hidden and tragic lives

The focus groups all started out by addressing the question of whether or not sexuality and gender identity were issues discussed in their workplaces. The almost unanimous answer in almost all cases was “no”. Many of the participants had encountered sexual relationships and sexual expressions in dementia care and some had worked more actively to affirm the sexual health of people with dementia. However, this had rarely involved experiences of or discussions of same-sex relationships or expressions.

That’s not something you notice, same-sex attraction or anything like that in the care home. (FG1)

It hasn’t really been an issue, something we’ve talked about, no. (FG3)

It’s surprisingly seldom that I’ve come across anyone [in need of care] who has been openly lesbian or gay. It’s strange really, because there are many LGBTQ people, that I haven’t met more of them. (FG4)

However, the absence or invisibility of LGBTQ people was not linked to dementia care practice. Instead it was repeatedly explained as resulting from generational belonging, an interpretative repertoire which was very pervasive in the focus groups. People who needed dementia care were positioned as part of a generation who had been forced to conceal their non-normative sexualities and gender identities, as seen in this interaction in focus group three:

IP 1: I’m not saying this to defend [myself] but I think that it has to do with the residents in or care homes they, haven’t been…

IP2: Open.

(FG3)

Participants in all five focus groups reflected on the historical contexts of shame, oppression, stigma and criminalisation of homosexuality and transsexualism which had restricted the possibilities of being open. This was sometimes addressed in temporal terms, that the people they cared for belonged to “the old times” or were “born in the wrong time” which was dominated by “old values”, which meant they had been forced to conceal or hide their sexual orientation. In some cases, concealment was linked both to time and place. Two of the focus groups were conducted in smaller cities and the participants there suggested that their regions were more intolerant and backward than the capital Stockholm and other bigger cities.

People in need of dementia care were understood to hold “old values”, an attitudinal position that was felt could have an impact on the interactions between residents in care homes. For example, one of the assistant nurses suggested that it was safer for LGBTQ people not to be open in dementia care due to the potentially negative attitudes from other people with dementia.

This generation that we are working with right now have a hard time accepting or respecting …so it’s better not saying anything. (FG5)

Care workers argued that expressions of intolerance from other residents were particularly linked to the dementia care home setting, which they described as a context prone to conflicts. According to the care workers, residents’ close living arrangements, combined with brain damage or reduced mental capacity, often led to lowered inhibitions

It’s more of a problem that the older [residents] are aggressive and give each other a hard time. You can’t deviate – there would be a lot of bashing, if someone deviated. That’s harder to handle really. (FG3)

Overall, LGBTQ people with dementia were recurringly positioned as having lived tragic lives that had involved concealment. In some focus groups same-sex sexual orientation was discussed in parallel to hidden traumas from the past such as incest or domestic violence, all of which were sensitive topics that could arise in care contexts as dementia illness progressed. This discursive linking of queer lives with tragedy, unhappiness, and other negative affects is extensively discussed in queer theory (see e.g. Ahmed, 2010; Goltz, 2010; Love, 2007). However, specific to our research is how LGBTQ people living with dementia were positioned as not only being trapped in the old times but also as having arrived at an *end station* – where dementia illness meant there were “no possibilities” remaining for living a queer life. For example, this association is reflected in the discussion of one of the vignettes in focus group three about how to deal with the possible hidden lesbian or bisexual life of a woman with dementia.

IP1: If she’d lived as a hidden lesbian, it’s really sad that she didn’t dare to come out of the closet. But she doesn’t seem to feel bad...the story doesn’t [reveal] – that there is an anguish or anxiety there….

IP2: Or she might feel bad. But how do you help a person with dementia who has been living with this restraint all her life? You know, life’s short, really short, and you’ve been living with this restrained life. And then suddenly you’ve got dementia. Is it really possible to help a person out of –

IP3: The closet.

IP2: Do you see what I mean? To actualise this, that’s not possible, that’s not my…

IP1: No, we’ve had people like this. Those who you’ve felt, apparently, like you were discussing before, that they would rather have wanted to be with a man. It’s not possible. There’s no way forward. […] People with dementia, you can’t argue with them in a logical way. It’s not possible to tell this person [with dementia]: “look, if you’d prefer to have been with a man, that’s completely fine, just let go of all of your inhibitions and live out your sexuality”. That’s not possible for this person. (FG3)

As seen in this extract, people with dementia are deemed to be “stuck in the closet” and invisible. Visibility is premised on their own willingness and capacity to come out; because their capacities are now diminished, their time for coming out is understood to have “passed”. The interpretative repertoire of generational belonging in combination with a dementia illness is thus used to explain the continued silence and invisibility of LGBTQ people in dementia care.

Notably, the invisibility of LGBTQ becomes, by means of this interpretative repertoire of generational belonging, not an effect of care practice per se. Instead invisibility is located in the people with dementia who are assumed to have lived repressed and closeted lives. This repertoire, albeit inadvertently, takes away the responsibility from care workers for affirming or making LGBTQ people with dementia visible in the care context. There were some exceptions and challenges to this repertoire. For example, one specialist nurse who had been actively involved in creating a sexual health policy in the municipality where she worked engaged in some critical self-reflection during the focus group when she asked rhetorically, “is it me who has been unwilling to see?” The tendency to locate invisibility in generational belonging was more pervasive, however, and was also linked to understandings of sexuality and gender identity as sensitive topics, as we will go on to discuss next.

### “You don’t know how to ´tiptoe through the jungle’”: Sexuality and gender identity as sensitive topics

Overall the participants in the focus groups in various ways engaged in speech that signalled tolerance. It was seen as desirable for LGBTQ people to be able to be open in care, findings that are in line with existing international research (see for example Simpson et al., 2018; Villar et al., 2015). Still, these issues were also understood as sensitive and private topics which made them difficult to approach.

LGBTQ – it’s still rather taboo, how to speak about and approach it…. You don’t know how to ´tiptoe through the jungle´ in order not to hurt or label. (FG3)

Understandings of sexuality and gender identity as private issues meant that asking questions about this was regarded as not always appropriate. Some participants raised the right to integrity when they said “we don’t have to know everything”. But there was also a concern, as the quote above indicates, that asking questions about gender identity or sexuality would result in “labelling” or “hurting” the people they cared for. This understanding seemed to go back to the issue of generational belonging. It was assumed that people with dementia had never been open and that they would therefore always be unwilling or ashamed to be associated with an LGBTQ identity.

The topic was also seen as a particularly delicate one to raise with people with dementia illnesses. Some focus groups saw them as particularly vulnerable. People with dementia were positioned as “sensitive”, where “things could escalate quickly”, and as “easily getting caught up in their emotions”. Starting discussions or evoking memories or experiences about gender identity or non-normative sexuality was thus regarded by participants as something that could potentially upset the people they cared for, which was in turn based on the underlying assumption that non-normative sexual lives had been unfulfilled and accompanied by tragedy or grief. One group talked about how a getting dementia diagnosis was a “tough experience” that raised a lot of emotions in general. One of the nurses we interviewed argued that in this situation raising a gender-neutral question such as “do you have a partner?” could cause some people to feel their sexuality was being exposed at a vulnerable point. For many of the participants, ensuring the well-being of people with dementia was regarded as central to their professional ethos. Raising these issues would potentially evoke negative feelings that were not conducive to well-being.

Understandings of sexuality and gender identity as sensitive topics figured in all of the focus groups. Still it varied in terms of what was understood as sensitive. Was it a sensitive topic for the people with dementia? Or was it rather, as suggested by some of the care workers interviewed, a sensitive topic for professionals in dementia care? For example, one of the occupational therapists remarked, in a discussion of the planning of care and the role of municipal care managers, that “everyone may not be comfortable raising these questions either”. An assistant nurse described some of her colleagues as having a “giggly attitude” when it came to these questions. These different understandings point to the presence of competing interpretative repertoires with “sensitivity”, one repertoire positioned people with dementia as sensitive whereas the other rather positioned it as a sensitive topic for care workers.

### “We’ve progressed”: the promise of generational shift and new visibility

While the interpretative repertoires of generational belonging and of sexuality and gender identity as sensitive topics had the effect of maintaining invisibility and silence in dementia care, there were also articulations of expected changes to come. Participants referred repeatedly to a “generational shift” under way in the healthcare sector. The next generation would soon enter dementia care, both as care workers and as receivers of care. This would allow for greater sexual visibility and more opportunities for openness. All of the focus groups spoke about changes in society, that “we’ve progressed” and become more tolerant, and that LGBTQ identities were becoming increasingly visible and normalised. These changes would have a knock-on impact on dementia care:

When my generation grow old I think it’s gonna be easier to tell [about your sexuality or gender identity]. It’s going to be more of a natural thing, that it’s included in these life story reviews, cause we’re allowed to marry same sex partners and there’s not so much shame in it. (FG1)

Although the invisibility of LGBTQ in dementia care was rarely understood as the effect of prejudices and the non-tolerant attitudes of care workers, younger generations were positioned as the potential for a different and more inclusive future. “So, when future generations are going to work and care for us,” said one nurse, “their views will be entirely different“. These kinds of articulations functioned to construct change in dementia care as part of a wider societal change that was already on its way. In effect, these articulations functioned as signposts to indicate that no immediate interventions were needed in today’s dementia care because the solution was already on its way with the coming generation.

The only potential issue that the care workers in our study suggested might threaten this new and more tolerant future in dementia care was the attitudes and values of the dementia care workers who had migrated to Sweden:

I understand that it’s going to be a bit of a challenge for us, we have quite a few [among the staff] who are not born in Sweden, with a different religion. Homosexuality is accepted in our society. (FG1)

This understanding of Sweden as a tolerant and progressive country in contrast to other places surfaced in several of the focus groups. However, few interviewees claimed that they had actually encountered negative attitudes or explicit homophobia among their colleagues with migrant backgrounds. Instead it was articulated in terms of supposition: “I would imagine. We haven’t talked about it”. These articulations reflect a wider so called *homonationalist* discourse where Sweden seen as LGBT-friendly, tolerant and progressive while migrant Others are understood as intolerant and threats to openness (Kehl, 2020; Polkov, 2024). The opinion was expressed that there was a need for education within dementia care, particularly for care workers from migrant backgrounds. The participants in our focus groups who were from migrant background themselves showed a clear awareness of this homonationalist discourse. On several occasions these participants made a conscious point to say that “treating everyone equally” and “showing respect regardless of gender or sexuality” was essential to dementia care practice. This suggests that being seen as a good *Swedish* care professional was strongly linked to articulations of equal treatment and LGBTQ-friendliness.

### Dementia as bringing out true gendered and sexual selves – or “it could have been a fantasy”?

Although the focus group participants had few experiences of caring for people with dementia who were open with their LGBTQ identities, some of the care workers had experienced expressions of same-sex desire or gender nonconformity (in particular cross-dressing) among the people with dementia. The interpretative repertoire the participants drew upon in this case was one which posited dementia as a loss of inhibition or restriction, as illustrated in the extract here from focus group five.

IP1: So when this man started to get dementia and lost his wife… Then it emerged... that he felt more like a woman than a man. And that it had been like that all along, even with his wife […]

IP2: It could be that these inhibitions disappear with the illness. You become yourself.

That ”one’s core” or “inner feelings” emerge as dementia progresses was a repertoire that was consistent throughout all of the focus groups. This discourse also reflected understandings of sexual orientation and gender identity as consistent and inherent (Silverman & Baril, 2021). The care workers in our study thus regarded dementia illnesses as something that could cause the revelation of previously concealed sexualities or non-normative gender identities or expressions. However, this exposure of gender nonconformity was understood as an effect of illness and as such was involuntary. It was not read as a positive sign of openness and “coming out”. Two of the focus groups also framed these revelations, in cases where people lived in heterosexual relationships, as “difficult care situations”; “when his wife comes by, how do I explain this?” one assistant nurse commented.

Interestingly, however, the repertoire of dementia as bringing out one’s true sexual and/or gendered self co-existed with another contradictory repertoire. In this people with dementia were positioned as non-accountable and expressions of same-sex desire were seen as inauthentic and part of the temporary confusions and fabulations that frequently occur among people with dementia. For example, in focus group three, one nurse spoke of a male resident she had encountered in dementia care who she described as “very confused, almost psychotic”.

And he started telling us stories about his sexual orientation. He’d been in Africa and had lots of relationships down there. But I don’t know if it was true or not. But you noticed later that he was drawn to men. He wanted to sit next to [men] at lunch and breakfast and all that, not women. So, it might have been true. But you never know, cause he was seriously confused and had…well, stories…

In this extract the matter of veracity figures as significant. Were these stories things the man had *actually* experienced or were they mere hallucinations created by his dementia? Although he displayed an orientation/desire towards men in the care setting, the truthfulness of his accounts becomes a central point for the nurse describing the situation.

Similar deployments of ‘truth versus fantasy’ occurred in other focus groups, particularly when discussing the vignettes. The stories of lesbian and gay people with dementia were met with comments such as: “we don’t know if this was true or not, it might just have been something she wanted to try”; “it could have been a fantasy”; “how much of this is wish and fantasy and what has he really been experiencing”; and “she might have been fabulating, it might never have happened”. This repertoire of queer sexuality in dementia as inauthentic is not isolated. It resonates with wider discourses on sexual expressions in dementia as inauthentic, unintentional and beyond control (see further discussions in Sandberg et al., 2020). Moreover, there is a long history of positioning queer sexualities as childish, immature and undeveloped (Stockton, 2009), discourses which also figured in our focus groups. When discussing one of the vignettes, in which a woman with dementia recounts her memory of a queer teenage infatuation, the focus group participants framed this in terms of youthful sexual experimentation: “When you’re a teenager you want to try everything” and “Oh my god, you did lots of things in your late teens”. The memory was thus not accepted as being a true reflection of a lesbian identification but rather as a fantasy or a youthful “phase” which was not relevant to who this woman was today, living in dementia care.

In our study, therefore, dementia illness is used in ambiguous and contradictory ways to construct gender and sexuality. The interpretative repertoire of dementia as bringing out one’s true self co-existed alongside the repertoire where dementia illness causes confusion and makes people not accountable for their articulations of sexuality and gender identity which are not viewed as real or authentic expressions anyhow. None of these repertoires frame non-normative sexuality or gender transgression as the result of dementia, as is often the case in the medical literature (Marshall et al., 2015; Sandberg et al., 2020). Still, dementia illnesses were used discursively to both validate the existence of LGBTQ people with dementia and to sustain heteronormativity by questioning and invalidating same sex desires or relationships.

### ”It doesn’t matter if it’s a teddy bear, a cushion, a woman or a man“: desexualising intimacy in dementia care

The questioning of the authenticity of queer sexual expressions among people with dementia reflects a form of unintelligibility of queer sexuality in dementia care. This unintelligibility was also manifested in how same-sex intimacies and touch were understood among the care workers in our study. Intimate touch, particularly between women, was consistently described and understood as non-erotic. For example, one assistant nurse in focus group four described one Christmas Eve in a dementia care unit where she worked:

And we found these little old ladies. The first thing we noticed was a basket with the Christmas roast that they had taken out of the fridge in it. And there they were, these little old ladies in one of the ladies’ beds, holding each other, being really happy, and it was really cozy. And I never remembered that we found it strange in any way or sexual… But they liked each other a lot and were holding and hugging each other…

This example positions these women with dementia as not only confused (putting the Christmas roast in a basket) but also as infantilised. They are “little old ladies” whose intimacy is harmlessly “cute” with no “strange” or purposeful erotic desire underpinning the incident. This example reflects a wider interpretative repertoire of desexualised intimacy in dementia care in our study. Touch was described in non-sexual ways and as particularly significant for people with dementia, who often had what they called “skin hunger”. Touch was articulated in terms of a basic human need rather than linked to sexual orientation, as seen in the two quotations below:

It doesn’t matter if it’s a teddy bear, a cushion, a woman or a man, it’s enough to be able to touch something because the need for intimacy is really great, gender is really not significant – or whether it’s a cushion. (FG1)

It doesn’t matter if it’s a cuddly toy, a man or a woman she touches. (FG2).

Similarly to the Christmas Eve example, these quotations reflect understandings of touch when living with dementia as asexual and childlike, with references to cuddly toys and teddy bears. That it was women who figured in these examples was also notable and not surprising. Same-sex desire and touch between women is often made invisible and interpreted in asexual terms, especially as they age (Hafford-Letchfield, 2021; Traies, 2016). This desexualisation was also intertwined with a heterosexualisation of women with dementia. Although the vignettes were presented within a project on LGBTQ and dementia, when intimacy and relationships between women were described, they were frequently commented on in terms of platonic friendship:

Maybe they’re just female friends. They’re friends and they want to be together. They feel secure together. (FG 5)

Touch and physical intimacy were understood as more common and normal between women in general, and were not necessarily seen as a sign of lesbian sexuality. Intimacy between women in dementia care was instead seen as a “cosy” or “comforting” part of friendship.

Although women with dementia were to a great extent heterosexualised and desexualised in the focus group discussions, interesting shifts started to occur when, during the course of the interviews, the participants started to challenge their own heteronormative interpretations of “non-erotic female friendships” in dementia care. This was particularly evident in one of the focus groups when discussing one of the vignettes, one of the assistant nurses started to reflect on her understanding of the intimate relationships she had encountered in dementia care:

When reading this… I’ve been in situations where women have been very close. And you’ve just seen them as friends. I never saw anything else…. But they were really like…almost like love. And that could have been… They might not get into bed, but you never know. We’ve got two women in particular – they enter each other’s rooms. Lock the doors. And you don’t know, you think…. So I don’t really know – if we’re the ones who don’t see...it could be like that. Why not…You ask as if we’ve had experiences [of caring for LGBTQ people with dementia]. You don’t know sometimes... (FG 4)

This assistant nurse spoke with considerable hesitation and uncertainty, likely signalling conflicting interpretative repertoires and ways of positioning intimate relationships between women with dementia. On the one hand there is the repertoire that heterosexualises and desexualises the intimacies between women with dementia in care settings. On the other there is an emerging repertoire of heteronormative ignorance or blindness in the focus groups which suggests the potential for a discursive shift.

## Concluding discussion

In this article we have explored discourses on gender identity and sexuality in everyday dementia care practice, focusing specifically on the articulations of Swedish dementia care workers. Our findings point to a pervasive heteronormativity. Sexuality and gender identity are rarely topics of discussion in dementia care settings and expressions of same-sex desire and queer intimate relationships are interpreted in desexualised terms as friendships, the need for comfort, or “skin hunger”, particularly when it came to the repertoires surrounding intimacies between women. Although dementia was seen as bringing out one’s true gendered and sexual self, expressions of non- normative sexualities were understood in terms of the fantasies and fabulations which are the result of dementia and not necessarily as authentic or truthful expressions. Such interpretations serve only to reinforce still further heterosexuality as the sexual norm.

People with dementia were positioned as belonging to a generation where LGBTQ identities had been stigmatised and repressed and LGBTQ people with dementia were understood as having lived tragic, closeted lives. We do not dispute the fact that the life histories of older LGBTQ people, including those living with dementia, have involved considerably silencing, discrimination and oppression; this is indeed supported by existing research, including our own (Siverskog, 2023; Smith et al., 2024; Fredriksen-Goldsen, 2018). However, an effect of this repertoire is to ascribe the invisibility of LGBTQ people to their own unwillingness or fear to be open – and not to the care practices they encounter per se (compare Villar et al., 2022). There was no reflection on the fact that dementia may involve particular challenges in the articulation of one’s self and one’s past and that it may make it difficult to assess with whom it is safe to be open (Barrett et al., 2015).

Although Swedish dementia care work is guided by legislation and policies that are person-centred and based on equal treatment, the care workers in this study did not see it as one of their responsibilities to enable LGBTQ people to feel able to share their queer life histories nor to provide a variety of gendered expressions. In fact, explicitly addressing gender and sexuality in the care context was seen as sensitive, potentially hurtful or a form of negative labelling. This seems to rest on the premise that LGBTQ identities were always negatively perceived and that these identifications had been hidden personal tragedies. While not so explicitly articulated, people with dementia seemed to be regarded as particularly vulnerable; evoking discussion of sexual orientation could thus be detrimental to their wellbeing. That LGBTQ people with dementia could have lived happy lives with deeply meaningful relationships and where sexuality or gender identity had been central to their sense of self, or even a source of wellbeing and pride, was never considered in the focus groups. This is particularly striking when juxtaposing it to the interviews we have conducted with LGBTQ people with dementia living in residential care, whose life experiences clearly included openness, pride, pleasure and happiness (Sandberg & Siverskog, 2024).

Our findings resonate with the wider literature on sexuality and gender identity in eldercare, but there were also specificities in terms of how dementia was employed to further desexualise people in care and to sometimes delegitimise expressions of non-normative sexuality and gender expression as fantasies or confusion. Moreover, because dementia illness was understood as bringing out a true self or an inner core, the result for care workers seemed to be inactivity; if sexual orientation and the gendered self would reveal itself eventually, the implication was that no interventions to challenge heteronormativity were seen to be necessary.

Clearly the care workers in our study had the best of intentions and worked with the well-being of those they cared for in mind. It is in no way our intention to cast them in a negative light. Instead, we want to echo Wetherell and Potter (1988, p. 182) who state that “well-intentioned talk can have reactionary consequences” and that ideological effects are often not the same as the motivations of the individual speakers. In this case heteronormativity persisted via the repertoires that the participants employed, although this was likely not what they aspired to (in most cases). As argued by Simpson et al. (2018, p. 871) “goodwill and reflexivity are necessary but not sufficient conditions for more collective forms of good practice required to secure equality of outcomes”. In some cases the best of intentions may in fact form obstacles to change and obscure lack of action.

In our study change and progress was understood as something that would inevitably emerge from a generational shift. Younger people who would enter the dementia care system, both as care workers and as receivers of care, were assumed to be more progressive, open and tolerant. Their attitudes and values were positioned as the catalyst that would break the silence and increase the visibility of LGBTQ people with dementia. We would caution against such trust in ‘natural’ change over time and the reliance on the progressiveness of coming generations. Over the last decade the rise of anti-gender movements and the far-right, both inside and outside of formal politics, has resulted in a notable increase in anti-LGBTQ discourse in North America and Europe (ILGA, 2023; The RESIST Project, 2024). People living with dementia in need of care are particularly vulnerable in these illiberal times and the need for active staff training and the design of non-heteronormative care is thus urgently needed (see also Shippee et al., 2024).

Finally, we wish to discuss how openness figured in our study as a prerequisite for LGBTQ people with dementia to have their selves affirmed and sustained in care. As one of our interviewees said:

If you’re open to begin with, it’s gonna be a whole lot easier – it’s easier for people to know how to approach you. (FG 2)

We suggest that openness, which has been a pervasive Western discourse in relation to LGBTQ rights over the last half century, builds on what King (2016, 2023) terms cogno-normativity. This means that cognitive function is not only assumed but also regarded as central to personhood. Being able to articulate oneself or account for one’s sexual preferences – to “come out” – is thus understood as a pre-requisite for the recognition and affirmation of an individual’s subjectivity. However, people living with dementia may not be able to articulate their self in these cogno-normative ways. Formal care workers as well as significant others thus play a central role in sustaining the self, including an individual’s life history and relationships. This does not necessarily mean that care workers should help people to “come out” as this could result in forms of hypervisibility and unwanted “outing” (Sandberg & Siverskog, 2024; Kia, 2019). Instead we propose a challenge to heteronormativity in dementia care. This could entail formulating policies that mandate straightforward actions such as using gender-neutral terms when speaking about partners and being flexible with the kind of pronouns and gendered expression a person with dementia may wish to use (and which may sometimes change) (see Silverman & Baril, 2021). It could also entail learning more about queer subcultures and LGBTQ history in one’s local context, including for example the names of nightclubs and meeting places, so that conversations can be initiated which extend beyond the heteronormative majority culture. Moreover, as LGBTQ people have often relied on social networks, friendship groups and LGBTQ communities rather than biological kin, dementia care providers could also seek to affirm friendships more and establish stronger connections with local LGBTQ groups and organizations, to enable people to break the potential isolation and segregation when living with dementia.

Our study points to considerably heteronormativity in dementia care. However, we also found examples of competing interpretative repertoires where care workers in our focus group conversations effectively challenged their own heteronormative repertoires – this points to the potential for change.

## Note

1 Our project focuses on the experiences of LGBTQ individuals. Our study did not collect data on intersex individuals, although we use the acronyms LGBTI or LGBT when referring to the government reports and research articles that used these acronyms.

## Appendix A

**Vignette 1:** Louise, 82, has dementia and when you first meet her, she has been living as a woman since her late 50s. She has been open about her transgender identity to everyone she meets in elderly care, she is on hormone therapy and has changed her legal gender to female a few years ago. Louise likes to be well-dressed and wants the staff to help her put on her make-up every morning. Over time, Louise’s interest in make-up and clothes starts to diminish. She also talks less and becomes more introverted. When staff ask her if they are going to do her make-up, she just waves them off. One day when you arrive, you see that Louise has packed all her clothes and make-up into bags and is taking them to the rubbish room. When you ask her what’s going on, she doesn’t answer but resolutely goes and throws the clothes away. She then dresses only in a shirt and jacket trousers and when you address her as Louise she quickly corrects, ‘Louise who is it?’. She says her name is Karl-Erik, the name she was given at birth.

**Vignette 2: A**nita is 76 years old and has Alzheimer's disease. She has lived in the dementia centre for just over a year and you have good contact with her. You talk a lot about her interest in gardening and the allotment where she has spent a lot of time. Anita has lived as a single parent with two children and none of the staff have heard of any fathers of the children or any other close relationships that Anita has had. One day, while you're out in the care home garden looking at the roses that have just started to bloom, Anita starts to tell you about a memory. She tells you about a crush she had when she was in her late teens; a Gunilla with the most beautiful light blue eyes. She tells how they used to meet in secret and kiss in the park one spring. She is happy and excited when she tells you, you can tell that this is important to her. You listen and ask questions about what happened next, but Anita finds it difficult to continue the story, she starts talking about her daughter instead and Gunilla is soon out of the story. When you ask Anita again about Gunilla a few weeks later, she first looks at you in surprise, then she gets angry and says she doesn't know what you're talking about, that she never knew anyone called Gunilla. You wonder if it could be that Anita has lived as a hidden lesbian during her life and has now come out.

**Vignette 3: **Nils has been struggling to manage his daily life at home for some time due to his dementia. He has just turned eighty when he moves into the home. Some days are tough for him, his memory fails him and he has trouble finding words. Other days he is in a good mood and words flow. He likes to tell the staff at the centre about his life and the love affairs and erotic encounters he has had with other men throughout his life. After decades of living in secrecy about his sexuality, he is now living openly in his old age. It has been a relief, he says, to be able to be open about who he is. Sometimes he has travelled to other cities to go to gay clubs and he talks about the incredible atmosphere there, being in a room full of other gay men, flirting, and maybe meeting someone to go home with. Lately, he has also been using the internet a lot as a way to connect with other men. He talks about how exciting it was to write to someone online and then meet up in person. Soon after moving into the centre, the staff notice that Nils seems to become isolated. He doesn't get any visitors and he expresses that he feels lonely and misses socialising, especially contact with other gay people. He brings his computer to the centre, but has not been able to connect to the internet and has therefore lost many of the contacts he used to have. He, who used to be a very sociable person, also feels a bit insecure.

**Vignette 4: **You are contacted by staff at a care home where two women have started a relationship. They tell you that the women, Stina and Hedvig, who have both lived there for about a year, have fallen in love with each other. Stina was widowed a few years ago, and no one knows much about Hedvig's previous relationships. Now they want to be close to each other all the time and often sit together. Talking, or just sitting quietly, close together. When Stina's relatives come to visit, she lies in bed with the other woman. The relatives get upset and think it is inappropriate. They become uncomfortable and want staff to help and keep the women separated.

**Vignette 5: **A new resident arrives at a care home who has felt like a woman all her life and has lived it out in certain contexts. But it is only in her old age that she has become more open in all contexts and has started hormone treatment. Arja, as she is known, had difficulty coping with everyday life on her own, which eventually led to her move to a care home. She has told the social worker about her trans identity before moving to the centre. She also told him that earlier in her life, because of her trans identity, she had ended up in violent situations, and that she had faced transphobia that had left a deep mark on her. These experiences have contributed to her not daring to be open for a long time and mean that she still feels fear in new contexts today. During the meeting with the social worker, she has mentioned what is important to her in care: that she should be seen as a woman and that those around her should use ‘she’ and the name she wants. She has changed her name, but not her legal gender/personality number, and although she started hormone treatment in her later years, her voice and build mean that her trans identity is often visible to others. Now that she has moved into residential care, one of the other residents who also has dementia is aggressive and rude to her when they are in the communal areas and consistently uses the wrong pronouns.

**Vignette 6: **Liv, who is in her 80s, is moving to a care home. She was married to a man earlier in life and has children from previous marriage. When a life story is collected after moving to the home, Liv tells us that in her old age she met another woman with whom she fell in love. They have had a close relationship in recent years. The woman also comes to the centre and visits her regularly. However, she has not told her children or grandchildren about the relationship and she does not have the courage to do so. She says she is worried about what will happen because her memory and cognitive abilities are changing rapidly. She thinks a lot about what will happen if and when others will be involved in making decisions about her care. She would like it to be her partner, but she knows that her children would like to be involved in such decisions.
